# Retraction: The impact of agricultural water salvation investment on economics development: Evidence from Eastern China

**DOI:** 10.1371/journal.pone.0347257

**Published:** 2026-04-15

**Authors:** The PLOS One Editors

The *PLOS One* Editors retract this article [1] due to concerns about:

- Non-compliance with PLOS policies on authorship.
- Text overlap with a previously published article [2] which does not share authors with [1].

All authors either did not respond directly or could not be reached.
